# Prediction of early postoperative atrial fibrillation after cardiac surgery: is it possible? The value of interatrial conduction time for the prediction of early postoperative atrial fibrillation using intra-operative transoesophageal echocardiography

**DOI:** 10.5830/CVJA-2011-010

**Published:** 2012-02

**Authors:** M Karaca, MI Demirbas, S Biceroglu, H Yilmaz, A Çevik, Y Cetin, Murat Arpaz

**Affiliations:** Department of Cardiology, Atakalp Heart Hospital, Izmir, Turkey; Department of Cardiology, Atakalp Heart Hospital, Izmir, Turkey; Department of Cardiology, Atakalp Heart Hospital, Izmir, Turkey; Department of Cardiology, Atakalp Heart Hospital, Izmir, Turkey; Department of Anesthesia, Atakalp Heart Hospital, Izmir, Turkey; Department of Anesthesia, Atakalp Heart Hospital, Izmir, Turkey; Department of Cardiovascular Surgery, Atakalp Heart Hospital, Izmir, Turkey

**Keywords:** atrial fibrillation, interatrial conduction time, cardiac surgery, transoesophageal echocardiography

## Abstract

**Background:**

Postoperative atrial fibrillation is common after cardiac surgery. In this study, we aimed to investigate the value of interatrial conduction time for the prediction of early postoperative atrial fibrillation, using intra-operative transoesophageal echocardiography.

**Methods:**

A total of 65 patients undergoing cardiac surgery in our hospital between January and March 2007 were prospectively evaluated, and 59 patients with sinus rhythm were included in the study. We performed transoesophageal echocardiography on all patients, and intra-operatively measured the interatrial conduction time, as recently described. The patients with episodes of atrial fibrillation during the post-surgery hospitalisation period were defined as group 1 and those without episodes were defined as group 2.

**Results:**

Mean interatrial conduction time was 74 ± 15.9 ms in group 1 and 54 ± 7.9 ms in group 2. The difference in interatrial conduction time between the two groups was statistically significant (*p* < 0.05). In this study we found a statistically significant interatrial conduction delay between the groups. Postoperative atrial fibrillation was more frequent in patients with a longer interatrial conduction time.

**Conclusion:**

Increased interatrial conduction time may cause postoperative atrial fibrillation and it can be measured intraoperatively by transoesophageal echocardiography.

## Abstract

Postoperative atrial fibrillation is a frequent complication, occurring in 30 to 50% of patients after cardiac surgery.^1^ It is associated with an increased risk of morbidity and mortality, it predisposes patients to a higher risk of stroke, requires additional treatment, and increases the costs of postoperative care.^2^,^3^

There are many clinical risk factors for developing postoperative atrial fibrillation, including age, gender, obesity, hypertension, diabetes mellitus, low left ventricular ejection fraction, hypoxia, chronic pulmonary lung disease, and left atrial size and diameter.^4^,^5^ In the study by Straus *et al.*, important peri-operative factors for the development of atrial fibrillation were: longer extracorporeal circulation, increased dose/number of inotropic drugs, blood transfusion, and elevated postoperative white blood cell count.^6^

Prolonged interatrial conduction time has been reported in patients with paroxysmal atrial fibrillation.^7^ Interatrial conduction delay may be an important parameter for the development of atrial fibrillation after cardiac surgery and it can be measured as accurately by transoesophageal echocardiography as by invasive electrophysiological methods.^8^

The prediction of atrial fibrillation may reduce postoperative complications and hospitalisation time after cardiac surgery. Previously published studies investigating the value of echocardiography or electrocardiography (ECG) for the prediction of postoperative atrial fibrillation indicate the need for other methods to predict early postoperative atrial fibrillation.

In this study, we aimed to investigate the value of interatrial conduction time for the prediction of early postoperative atrial fibrillation using intra-operative transoesophageal echocardiography (TEE).

## Methods

Sixty-five patients undergoing cardiac surgery in our hospital between January and March 2007 were prospectively evaluated. Patients in sinus rhythm and with no known history of episodes of atrial fibrillation before surgery were included in the study. Patients were followed for the occurrence of atrial fibrillation during the hospitalisation period. We collected the clinical data with the permission of the local ethics committee.

All clinical characteristics of patients were noted (hypertension, diabetes mellitus, age, gender, indications for surgery, etc). After discharge from the postoperative care unit, all patients were followed for the occurrence of episodes of atrial fibrillation using ECG holter monitoring, which was performed for all patients until discharge from hospital.

Transthoracic echocardiography was performed in all patients before surgery. Left ventricular function was evaluated and the diameters of the cardiac chambers were measured. The diameters of the left ventricle and left atrium were measured from the parasternal short-axis view. The left ventricular ejection fraction was calculated by the Simpson method.

Intra-operative TEE was performed on all patients included in the study after the induction of anesthesia. Interatrial conduction times were measured as published previously.^8^

**Fig. 1:**
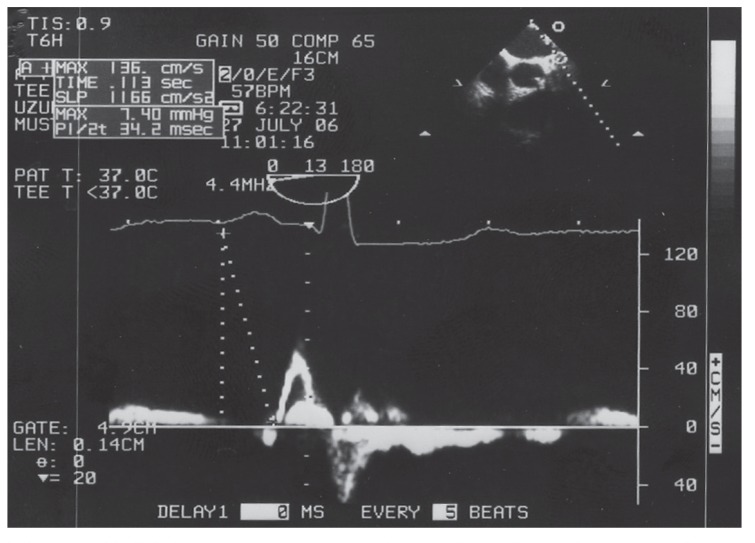
P-LAA was measured as the time interval from the initiation of the P wave on surface ECG to the start of the left atrial appendix ejection flow demonstrated by transoesophageal echocardiography.

The time between the origin of the P wave on the surface electrocardiogram and the left atrial appendage ejection flow (P-LAA) was measured by TEE and defined as interatrial conduction time (Fig. 1). The cross-clamp time was also reported. Patients with at least one atrial fibrillation episode after surgery during the hospitalisation period were placed into group 1 and the patients without episodes were in group 2. We compared the interatrial conduction times between these two groups.

## Statistical analysis

Data are expressed as mean ± SD for continuous data and as number and percentage for categorical data. A *p*-value < 0.05 was considered significant. Differences between groups were compared with the Student’s *t*-test on SPSS.

## Results

A total of 59 patients in sinus rhythm were included in the study. Thirty-nine of the patients were operated on for coronary artery disease only, and 14 for valvular heart disease only. Six of the 59 patients were operated on for both coronary artery and valvular heart disease. Atrial fibrillation was observed in 22 patients (37%) in the follow-up period. Baseline clinical characteristics were not statistically different between the two groups.

Intravenous and oral amiodarone was initiated for patients developing post-operative atrial fibrillation. In two of the cases, electrocardioversion was necessary for maintaining sinus rhythm. All of the patients were discharged from the hospital in sinus rhythm. The frequency of occurrence of atrial fibrillation was similar to that reported recently. The clinical properties are summarised in Table 1.

**Table 1:** Clinical Properties Of The Patients

	Group 1* (n = 22)	Group 2** (n = 37)
Mean age (years)	66 ± 5	66 ± 6
Male/female *(n)*	10/12	23/14
Hypertension, *n* (%)	16 (72)	21 (56)
Diabetes mellitus, *n*/(%)	8 (36)	16 (43)
Smoking, *n* (%)	13 (59)	19 (51)
Mean follow-up time (days)	6.7 ± 1.0	6.6 ± 0.8

*Patients with atrial fibrillation episode in follow up. **Patients without atrial fibrillation episode in follow up.

Atrial fibrillation has been detected more frequently in females than males (46 vs 30%). In our study, hypertension was more common in patients with atrial fibrillation than those without but the difference did not reach statistical significance. Additionally, patients with atrial fibrillation were older, although the difference did not reach statistical significance. Valvular heart disease seemed more likely to cause atrial fibrillation than coronary artery disease within the postoperative period (Table 2).

**Table 2:** Indications Of The Surgery

	Group 1* (n = 22)	Group 2** (n = 37)
Coronary artery disease *(n)*	13	26
Valvular heart disease *(n)*	12	2
Both coronary artery and valvular heart disease *(n)*	5	1

*Patients with atrial fibrillation episode in follow up. **Patients without atrial fibrillation episode in follow up.

The echocardiographic properties are summarised in Table 3. The mean left atrial diameter was slightly larger in group 1, and the mean left ventricular ejection fraction was slightly reduced but these values were not statistically significant.

**Table 3:** Echocardiographic Properties Of The Patients

	Group 1* (n = 22)	Group 2** (n = 37)	p-*value*
Left ventricular diastolic diameter (mm)	52 ± 6	51 ± 6	NS
Left atrial diameter (mm)	39 ± 5	38 ± 4	NS
Left ventricular ejection fraction (%)	46 ± 8	47 ± 7	NS

*Patients with atrial fibrillation episode in follow up. **Patients without atrial fibrillation episode in follow up. NS = not significant.

Mean interatrial conduction time was 74 ± 15.9 ms in group 1 and 54 ± 7.9 ms in group 2. The difference in interatrial conduction time between the two groups was statistically significant (*p* < 0.05). Mean cross-clamp time was 32.2 ± 9.5 minutes and there was no statistically significant difference between the groups.

## Discussion

Postoperative atrial fibrillation causes prolongation of hospital stay and it is a frequent complication occurring in 30 to 50% of the patients after cardiac surgery.^9^ There are many defined clinical risk factors for atrial fibrillation following cardiac surgery. Previous studies have shown that age and hypertension are important risk factors for atrial fibrillation.^10^ However in our study, there was no statistically significant difference between the two groups, possibly because our study population was too small to detect a difference.

Recent clinical trials have investigated echocardiographic parameters for the prediction of postoperative atrial fibrillation. P-wave duration on surface ECG and P-wave dispersion were found to be important and easily obtainable parameters for the prediction of postoperative atrial fibrillation.^11^,^12^ Prior to this, Stafford *et al.* found that signal-averaged P-wave duration was a better predictor of atrial fibrillation after coronary artery bypass grafting (CABG) than standard echocardiographic criteria.^13^ In our study, we did not investigate any electrocardiographic parameters for prediction.

Transthoracic echocardiography is a useful technique for the prediction of postoperative atrial fibrillation. In most of the recent trials, left atrial size and left ventricular systolic function were easily obtained for the prediction.^13^ Roshanali *et al.* investigated the importance of atrial electromechanical interval using transthoracic tissue Doppler echocardiography, and found it to be a valuable method for identifying patients vulnerable to post-CABG atrial fibrillation.^14^ Further clinical trials were necessary for the prediction of postoperative atrial fibrillation using tissue Doppler echocardiography.^15^ Interatrial conduction time can therefore be used for the prediction of postoperative atrial fibrillation.

Fuenmayor *et al.* found a new method for measuring interatrial conduction time, using transthoracic echocardiography. They simultaneously measured the time interval between the electrocardiographic P wave and the mitral a wave using transthoracic Doppler echocardiography and compared this with another more invasive method. They found similar results and concluded that transthoracic Doppler echocardiography combined with surface electrocardiography can be used for measuring the interatrial conduction time with a similar accuracy as other more invasive methods.^16^

Transoesophageal echocardiography has not frequently been used for the prediction of postoperative atrial fibrillation in recent clinical trials. TEE was however found to be a useful tool for measuring interatrial conduction time.^8^ In the study by Kinay *et al.*, a correlation between the interatrial conduction time and recurrence of atrial fibrillation was established. We therefore concluded that intra-operative measurement of interatrial conduction time by TEE could predict postoperative atrial fibrillation.^8^,^17^

In this study we found a statistically significant interatrial conduction delay in group 1. Increased interatrial conduction time may result in postoperative atrial fibrillation and it can be measured by intraoperative TEE.

Postoperative atrial fibrillation may prolong the hospitalisation period, particularly time in the intensive care unit, which may increase the risk of postoperative complications such as nosocomial infections. Using anti-arrhythmic agents for patients with prolonged interatrial conduction time before postoperative atrial fibrillation occurs could decrease the risk of postoperative complications.

## Conclusion

In this study we found that postoperative atrial fibrillation was more frequent in patients with longer interatrial conduction times. Measurement of interatrial conduction time by TEE may be a valuable method for the prediction of postoperative atrial fibrillation, and interatrial conduction delay is an important risk factor. We need more studies to define the cut-off point for interatrial conduction time.
